# Early Experiences of Image Guided Prostate and Pelvic Nodal Irradiation With Intensity Modulated Radiation Treatment in Localized Prostate Cancer

**DOI:** 10.4021/wjon436w

**Published:** 2012-02-19

**Authors:** Christine Ko, Holly Ning, Elena Lita, Deborah McNally, Bradford J Wood, Peter Choyke, Peter Guion, Sharon Smith, Axel Krieger, Kevin Camphausen, Anurag K Singh, Aradhana Kaushal

**Affiliations:** a Radiation Oncology Branch, National Cancer Institute, National Institutes of Health, Bethesda, MD, USA; b Urologic Oncology Branch, National Cancer Institute, National Institutes of Health, Bethesda, MD, USA; c Molecular Imaging Program, Center for Cancer Research, National Cancer Institute, National Institutes of Health, Bethesda, MD, USA; d Sentinelle Medical Inc Toronto, Ontario, Canada; e Department of Radiation Medicine, Roswell Park Cancer Institute University at Buffalo School of Medicine, Buffalo, NY, USA

**Keywords:** IMRT, Prostate cancer, Dose escalation, Radiotherapy, Toxicity

## Abstract

**Background:**

To present the early findings of a phase I clinical trial studying the use of intensity modulated radiation treatment (IMRT) to treat at risk pelvic and lower para-aortic lymph nodes in patients with high risk prostate cancer while escalating dose. Dose escalation was performed with a technique particularly aiming to limit the dose to surrounding critical structures.

**Methods:**

A total of 12 patients were treated with an IMRT plan that delivered 45 Gy to the pelvic lymph nodes, prostate and proximal seminal vesicles. This was followed by an image guided IMRT plan that delivered 9 Gy to the prostate and seminal vesicles and then an additional 21.6 Gy delivered to the prostate for a total dose of 75.6 Gy to the prostate. Gastrointestinal (GI) and genitourinary (GU) toxicity were recorded weekly throughout treatment and in follow up (range: 20 - 49 months).

**Results:**

At diagnosis, median age was 64, median PSA 15.5 (range: 5 - 103) and Gleason score ranged 7 - 9. The median dose to the bladder was 52 Gy, the median dose to the rectum was 53 Gy and the median dose to the small bowel was 26 Gy. During treatment, Grade 2 GU toxicity was noted in 3/12 (25%) patients and Grade 2 GI toxicity was noted in 2/12 patients (16%). At a median follow-up of 28 months, Grade 2 late GI toxicity was seen in 1/12 (8%) and late GU in 3/12 (25%) of patients. There were no acute or late grade 3 and 4 GU or GI toxicities.

**Conclusions:**

Our study shows the feasibility of using IMRT for pelvic and lower para-aortic nodal irradiation as the toxicities are low for the total dose that was delivered. This shows promise for reducing normal tissue doses, improving target control, and potentially allowing for additional dose escalation to the pelvic/lower para-aortic lymph nodes in our successive cohorts.

## Introduction

Prostate cancer is the most prevalent cancer among men and the third most common cause of death among men [1]. For many years clinicians have tried several strategies to improve outcomes in patients with localized prostate cancer. Patients with high risk prostate cancer [defined as a PSA > 20, ≥ cT2b, Gleason score ≥ 8] have received androgen suppression therapy (AST) in addition to definitive radiation therapy. There have been multiple randomized trials that have shown a benefit in biochemical control and some with an overall survival benefit to the combined use of AST and pelvic radiation therapy [2-6]. However, a different method to potentially further optimize outcomes is to escalate the dose of radiation. Multiple randomized studies have shown an improvement in progression free survival and failure free survival both in Northern America and in Europe when dose to the prostate is increased [7-14]. However, the limitation inherent to this technique is the normal tissue toxicity, particularly doses to the small bowel.

Intensity modulated radiation treatment (IMRT) has been used for dose shaping in prostate cancer, with what appears to be good clinical outcomes while respecting [deleted regarding] normal tissue toxicity [15]. IMRT may also allow dose escalation to be applied to the at-risk lymph nodes of high risk prostate cancer patients, as it may allow for sparing of the small bowel. A few institutions have attempted to use IMRT as a method of sparing bowel, the dose limiting structure of pelvic RT, while treating pelvic nodes in prostate cancer [16-18]. In a recent publication, researchers in Norway found that the use of IMRT improves target coverage and reduces normal tissue dose [order of these two phrases were swapped], which may correlate to an improved toxicity profile [19]. In Italy they have also looked into the dose-volume effect of radiation on bowel toxicity and they found that an IMRT approach drastically reduced the incidence of acute bowel toxicity [20]. In the UK, researchers found acceptably low bowel toxicity with dose escalation up to 55 Gy to the pelvis [18].

At our institution, we implemented a Phase I clinical trial evaluating the use of IMRT in the high risk patient group with the aim of escalating the dose to the pelvic lymph nodes. The primary objective was to determine the feasibility of treating pelvic lymph nodes with IMRT. The secondary objective was to evaluate long-term effects and toxicity following IMRT dose escalation to the pelvic nodes. In this report, we present our early experiences in target volume definitions and resulting dose distributions and toxicities.

## Materials and Methods

### Patient characteristics

Between February 2006 and May 2008, 12 patients were prospectively enrolled on an institutional review board approved Phase I study using intensity modulated radiation therapy (IMRT) to irradiate the pelvic and lower para-aortic nodes and the prostate and seminal vesicles in patients with high risk prostate cancer. Eligible patients were greater than 18 years of age and less than 90 years of age, ECOG performance status of ≤ 2, pathology report confirming adenocarcinoma of the prostate, risk of lymph node metastasis greater than or equal to15% as defined by the Partin tables [21] or biopsy proven positive lymph nodes, tumor must be visible on MRI, and with no history of prior surgery, radiation or chemotherapy for prostate cancer (with the exception of hormonal therapy). Exclusion criteria include cognitively impaired patients who cannot give informed consent, patients with metastatic disease beyond the pelvis, any contraindication to biopsies, any contraindication to having an MRI performed and any pre-existing and active prostatitis or proctitis.

### Treatment specifics

Procedures for simulation and planning were identical for all patients. Prior to CT simulation, patients had four gold fiducial markers (CIVCO, 1.2 x 3 mm fiducials (MT-NW-887-814) placed with the assistance of APT MRI: manipulator for access to prostate tissue under magnetic resonance imaging guidance (APT-MRI) [22]. Each patient was simulated 4 - 5 days after the fiducial marker placement to allow the fiducials to settle in the prostate. For simulation, patients were placed in the supine position with leg immobilization and a planning CT scan was performed with 0.3 cm spacing between slices. The CT scans extended from T12 to the upper third of the femur. Patients were asked to come to the simulation and daily treatment with a “comfortably full” bladder; to ensure similar daily bladder fullness, the patients were asked to drink 1 - 2 cups of water 15 minutes prior to their treatment. Patient setup was monitored daily using fiducial marker localization.

Using the Eclipse treatment planning system (Varian Oncology Systems, Palo Alto, CA), the treating radiation oncologist contoured three sets of clinical target volumes, CTV1, CTV2 and CTV3. CTV1 consists of prostate, seminal vesicle and pelvic lymph nodes, CTV2 is the sum of prostate and seminal vesicle and CTV3 is the prostate. The contouring of the prostate was aided by a fused MRI performed on all patients prior to the CT simulation. The pelvic lymph node contours for CTV1 were defined as the aorta 2 cm proximal to the bifurcation, the common, internal and external iliac [omitted “vessels are contoured”] (plus 2 cm concentrically) to the level of the most superior slice of the seminal vesicle [23]. PTV1 was this previously mentioned volume in addition to PTV2 and PTV3. PTV2 and PTV3 were determined by applying a total margin of 9 mm around the CTV2 and CTV3, except posteriorly, where a 5 mm margin was used.

The treating radiation oncologist also outlined the relevant organs at risk (OARs), including the bladder, rectum, and small intestines. The bladder was outlined from apex to dome and the rectosigmoid flexure was applied as the cranial limit of the rectum and the anal verge as the caudal limit. The small intestinal volume was contoured superiorly to the level of PTV1, including all identifiable small intestines. These volumes were based on the planning scan only and no attempts were made to account actively for the motion of these organs.

The radiation course for all patients consisted of an initial IMRT plan delivering 45 Gy to the PTV1 followed by an IMRT plan delivering 9 Gy to the PTV2 and then another 21.6 Gy to the prostate only for a total dose of 75.6 Gy to the prostate all delivered in 1.8 Gy daily fractions, five days per week. Intensity modulation was achieved using the sliding window method and most patients were treated with a five-field or seven-field technique using 6MV X-ray beams. All treatment planning and optimization was performed using Eclipse. Patients were treated on one of two Varian Clinacs (2100EX2999); both are equipped with MLC-120 multi-leaf collimators. One is equipped with EPID (electronic portal imaging devices) and the second is equipped with EPID and OBI (On Board Imager). Daily setup is performed by taking a pair of orthogonal images and performing a 2D/2D match for the pelvic fields and fiducial marker matching when treating the cone-down fields.

The optimization constraints were given to achieve these priorities: first, at least 95% of the PTVs receiving the prescribed dose; second, less than 25% of the rectum receiving 70 Gy; third, less than 40% of the dose getting 65 Gy and less than 10 cc of small bowel receiving 55 Gy. Patient specific quality assurances were performed prior to the patient’s treatment.

### Analysis of dose-volume histogram

Dose-volume histograms (DVHs) were calculated for all defined volumes (for example the PTVs, bladder, rectum, small intestine) from the sum plans in all twelve patients. In this paper we focus on analyzing the DVHs for the OARs rather than the PTVs.

### Toxicity scoring

The NCI Common Toxicity Grading Criteria and RTOG toxicity scoring system were used to grade lower GI and GU morbidity during and after the course of treatment [24]. Patients were scheduled for weekly assessments of symptoms during the course of radiation therapy by the responsible oncologist. Acute effects were scored weekly during treatment and during the scheduled follow-up visits. In general the GI or GU symptoms that needed medical prescriptions were scored as Grade 2 or greater toxicity.

## Results

### Patient characteristics

At diagnosis, median age was 64 (range: 50 - 74 years of age), median PSA was 15.5 (range: 5.7 - 103) and median Gleason score 8 (range: Gleason 7 - 9). The details of the patients’ characteristics are displayed in Table 1. The median follow-up time was 28 months (range: 17 - 49 months). Patients allowed on this trial were defined as high risk prostate cancers defined by the Partin tables (Partin 2001). All patients received neoadjuvant, concurrent and adjuvant hormonal therapy and pelvic, seminal vesicle, and prostate radiation therapy (RT).

**Table 1:** Characteristics of Study Patients

Characteristics	Value
Number of patients	12
Median age (years)	64
Age range (years)	50 - 74
Clinical stage	
T1	5
T2	6
T3	1
T4	0
Tx	0
Gleason sum	
≤ 6	0
7	2
8	5
9	5
10	0
PSA (ng/L)	
< 4	0
4 - 10	3
> 10, < 20	4
> 20	5

### DVH analysis

The median bladder volume is 126 cc, median bladder dose is 51.85 Gy and V60 bladder median was 22% range (16-41%), maximum dose of 79.04, and D40 54.49 Gy. The median rectal dose of 53.45; median max dose 77.72 Gy, median V65 15.05%, median V50 58.1%, median D25 60.11 Gy, median D50 52.26 Gy. The median small bowel dose is 25.8 Gy, median max dose 48.51 Gy, median D10 cc 47.18 Gy.

### Adverse effects

No Grade 3 or higher GI adverse effects were observed among the patients either during treatment or during follow-up appointments. During treatment, Grade 2 GU toxicity was noted in 3/12 (25%) patients and Grade 2 GI toxicity was noted in 2/12 patients (16%). At a median of 31 months, Grade 2 GU toxicity was seen in 3/12 (25%) of patients and Grade 2 GI toxicity 1/12 (8%) patients. There were no acute or late grade 3 and 4 GU or GI toxicities. Refer to Table 2 for details.

**Table 2:** Toxicity Associated With Treatment

Toxicity	Number of patients
Acute GU Grade 1, 2	8/12
Acute GI Grade 1, 2	10/12
Late GU Grade 1, 2	6/12
Late GI Grade 1, 2	5/12

**Figure 1:**
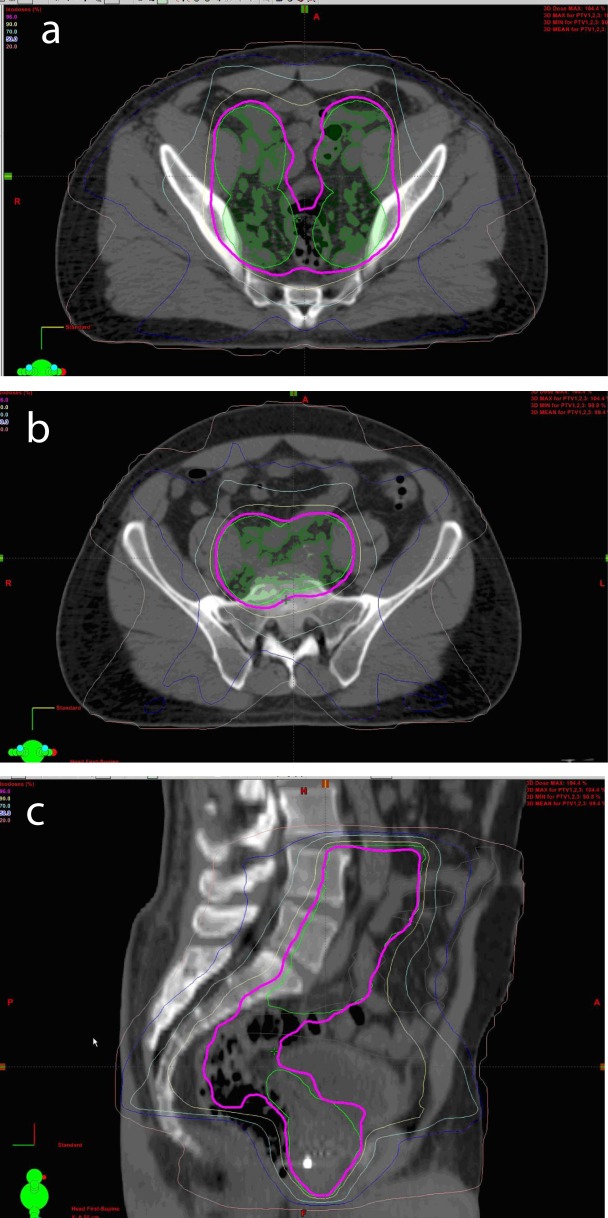
Axial View of Conformal IMRT Isodoses in Pelvis (a, b). Sagittal View of Conformal IMRT Isodoses in Pelvis (c)

All patients were able to complete the full course of treatment and were followed closely, one patient was take off the protocol as he subsequently moved out of country and was unable to be followed as per the predetermined schedule (Fig. 1).

## Discussion

While dose escalation to the prostate has shown to improve outcomes, dose escalation to the pelvis has not been successful, secondary to increased rates of gastrointestinal toxicity [25]. Our study and cohort of patients is particularly encouraging because of the low incidence of GI/GU toxicity as compared to currently published reports. An older study has proposed that less than 78 cm^3^ of the small bowel should receive more than 45 Gy [25]. In Norway they found that the use of IMRT could critically decrease the dose to normal structures compared to 3D CRT. With the use of IMRT, they were able to administer 50 Gy to the pelvis, prostate and seminal vesicles with an additional 20 Gy to the prostate and seminal vesicles with low rates of toxicity [19]. In the United Kingdom, they have one of the largest studies to date using IMRT to dose escalate to the pelvis to 50 Gy and 55 Gy with a total of 70 Gy to the prostate; the toxicity rates are reasonable given the dose to the pelvis. They also evaluated their patients based on the bowel that was contoured and found RTOG acute Grade ≥ 2 to be 38% and 51% for patients who had < 450 cc versus ≥ 450 cc bowel contoured [18]. Also of interest, this trial [deleted had] found that no one particular dose effect was apparent thus far for the 50 Gy and 55 Gy cohort as roughly the same numbers of patients had RTOG Grade 2 toxicity.

The RTOG 92-02 trial treated the pelvic lymph nodes to 45 Gy (no IMRT for the pelvic field) and 65 to 70 Gy to the prostate [5]. The EORTC 22,863 trial treated the pelvis to 50 Gy and 70 Gy to the prostate [26]. The toxicity outcomes are listed in Table 3 [5, 27]. For our study we found that for acute Grade 1 and 2, GU and GI side effects were relatively less than what was seen in these studies using hormonal therapy and treatment to the pelvis in addition to the prostate and seminal vesicles (Table 3). In the previously referenced studies, where patients received equivalent or lower total radiation doses, the rates of Grade 3 or 4 toxicities are low but present. Particularly notable is that in our study however, revealed no evidence of Grade 3 or 4 GU or GI toxicity in both the acute or late effect profile thus far.

**Table 3:** Comparison of GU and GI Toxicities in Selected TArials

Study	RTOG 92-02	EORTC 22863	UK study	Norwegian study	NCI study
Acute GU G 1, 2	529/758 (70%)		71/79 (90%)	36/43 (84%)	8/12 (67%)
Acute GU G 3, 4	25/758 (3%)	7%	1/79 (1.3%)	1/43 (2%)	0%
Acute GI G 1, 2	666/758 (88%)		74/79 (94%)	34/43 (79%)	10/12 (83%)
Acute GI G 3, 4	11/201 (5.5%)	5%	1/79 (1.3%)	0%	0%
Late GU G 1, 2	12.5% (Gr 2 only)	12.5% (Gr2 only)	2.5% (Gr 2)	20/43 (47%)	6/12 (50%), 25% (Gr 2 only)
Late GU G 3, 4	3.4%	3.4%	2.5%	1/43 (2%)	0%
Late GI G 1, 2	9.5% (Gr 2 only)	9.5% (Gr 2 only)	25%	22/43 (51%)	5/12(42%), 8% (Gr 2 only)
Late GI G 3, 4	0.3%	0.3%	1.3%	0%	0%

The results in Table 3 should be interpreted in the context of how CTV pelvic lymph nodes are defined in this trial and the previously referenced studies. The UK study, this CTV was defined as the uninvolved pelvic LN (obturator, internal iliac and external iliac chains bilaterally up to and including the common iliac region and pre-sacral nodes anterior to the first, second, and third sacral vertebrae) with no margin [18]. Our contouring of the LN groups is different: a radial 2 cm margin of the vessels (distal aorta, common, internal and external iliacs). This was based on a study that estimated that 94.5% of nodal failures were likely to be within 2 cm of these vessels. This study also demonstrates that 61% of the nodal failures were within 2 cm superior to the bifurcation and 10.2 cm inferior [23]. We, however, did not include the pre-sacral LN. The group from Norway had similar CTV to our group: 2.5 cm ring on the internal and external iliac vessels and did not include the pre-sacral LN [19]. At the time this Phase I trial was conceived, the current RTOG recommendations had not been available. We recognize that our nodal volumes differ from the current RTOG contouring atlas which recommends contouring from 7 mm around iliac vessels contouring out bladder, bowel, and bone; commence at distal common iliac vessels at L5/S1 interspace and to stop contouring at the top of the femoral heads; recommend treatment of presacral LN (subaortic only).

There has been controversy surrounding the role of pelvic RT in patients with a high risk of prostate cancer. There have been randomized studies to show the benefit of hormonal therapy in addition to RT (which included whole pelvic RT) but these did not address the benefit of elective whole pelvis irradiation [2-5]. There have been nonrandomized studies that address this question but with conflicting results [28-32]. While the initial results of RTOG 94-13 had been promising, the later follow-up failed to show an improvement in progression free survival [33]. The Groupe d’Etude des Tumeurs Uro-Genitales (GETUG) trial also showed no benefit for OS or PFS [34]. One of the possible reasons for this lack of benefit is postulated to be that a higher dose of radiation is needed to eradicate potential lymph node metastases. Our current study has moved on to the next cohort. Perhaps this dose escalation will address this theoretical concern of suboptimal pelvic nodal dosing.

Our results are interesting in that at this current follow-up, we have found that we can limit the dose to the small bowel, which has led to [delete “a decrease in both”] acceptable acute and late toxicities. In order to better assess the late term toxicity, we need a longer follow-up and investigate whether these results are sustainable for the long term. However, these current findings are encouraging that we can safely escalate to a higher dose. We adhered to the small bowel constraints as documented in the literature, but with longer follow-up these may be subject to modification. This Phase I trial has continued onto the next two cohorts where the lymph node groups receive 5040 cGy and 5400 cGy respectively, and we are hopeful that the side effect profile will be tolerable thus giving us the ability to reach higher doses to the pelvis with the use of image guided IMRT.

## References

[1] Jemal A, Siegel R, Ward E, Hao Y, Xu J, Murray T, Thun MJ (2008). Cancer statistics, 2008. CA Cancer J Clin.

[2] Bolla M, Collette L, Blank L, Warde P, Dubois JB, Mirimanoff RO, Storme G (2002). Long-term results with immediate androgen suppression and external irradiation in patients with locally advanced prostate cancer (an EORTC study): a phase III randomised trial. Lancet.

[3] Bolla M, de Reijke TM, Van Tienhoven G, Van den Bergh AC, Oddens J, Poortmans PM, Gez E (2009). Duration of androgen suppression in the treatment of prostate cancer. N Engl J Med.

[4] Pilepich MV, Winter K, Lawton CA, Krisch RE, Wolkov HB, Movsas B, Hug EB (2005). Androgen suppression adjuvant to definitive radiotherapy in prostate carcinoma—long-term results of phase III RTOG 85-31. Int J Radiat Oncol Biol Phys.

[5] Horwitz EM, Bae K, Hanks GE, Porter A, Grignon DJ, Brereton HD, Venkatesan V (2008). Ten-year follow-up of radiation therapy oncology group protocol 92-02: a phase III trial of the duration of elective androgen deprivation in locally advanced prostate cancer. J Clin Oncol.

[6] Roach M, Bae K, Speight J, Wolkov HB, Rubin P, Lee RJ, Lawton C (2008). Short-term neoadjuvant androgen deprivation therapy and external-beam radiotherapy for locally advanced prostate cancer: long-term results of RTOG 8610. J Clin Oncol.

[7] Pollack A, Zagars GK, Smith LG, Lee JJ, von Eschenbach AC, Antolak JA, Starkschall G (2000). Preliminary results of a randomized radiotherapy dose-escalation study comparing 70 Gy with 78 Gy for prostate cancer. J Clin Oncol.

[8] Pollack A, Zagars GK, Smith LG, Lee JJ, von Eschenbach AC, Antolak JA, Starkschall G (2000). Preliminary results of a randomized radiotherapy dose-escalation study comparing 70 Gy with 78 Gy for prostate cancer. J Clin Oncol.

[9] Sanguineti G, Agostinelli S, Foppiano F, Franzone P, Garelli S, Marcenaro M, Orsatti M (2002). Adjuvant androgen deprivation impacts late rectal toxicity after conformal radiotherapy of prostate carcinoma. Br J Cancer.

[10] Feigenberg SJ, Hanlon AL, Horwitz EM (2003). Androgen deprivation increases late morbidity in prostate cancer patients treated with 3D conformal radiation therapy [Abstract]. Int J Radiat Oncol Biol Phys.

[11] Michalski JM, Winter K, Purdy JA (2003). Toxicity following 3D radiation therapy for prostate cancer on RTOG 9406 dose level V [Abstract]. Int J Radiat Oncol Biol Phys.

[12] Kuban D, Pollack A, Huang E, Levy L, Dong L, Starkschall G, Rosen I (2003). Hazards of dose escalation in prostate cancer radiotherapy. Int J Radiat Oncol Biol Phys.

[13] Zietman AL, DeSilvio ML, Slater JD, Rossi CJ, Miller DW, Adams JA, Shipley WU (2005). Comparison of conventional-dose vs high-dose conformal radiation therapy in clinically localized adenocarcinoma of the prostate: a randomized controlled trial. JAMA.

[14] Zietman AL, Bae K, Slater JD, Shipley WU, Efstathiou JA, Coen JJ, Bush DA (2010). Randomized trial comparing conventional-dose with high-dose conformal radiation therapy in early-stage adenocarcinoma of the prostate: long-term results from proton radiation oncology group/american college of radiology 95-09. J Clin Oncol.

[15] Zelefsky MJ, Fuks Z, Hunt M, Yamada Y, Marion C, Ling CC, Amols H (2002). High-dose intensity modulated radiation therapy for prostate cancer: early toxicity and biochemical outcome in 772 patients. Int J Radiat Oncol Biol Phys.

[16] Cavey ML, Bayouth JE, Colman M Is dose escalation to the prostate feasible while treating the pelvic nodes with IMRT? [Abstract].

[17] Nutting CM, Convery DJ, Cosgrove VP, Rowbottom C, Padhani AR, Webb S, Dearnaley DP (2000). Reduction of small and large bowel irradiation using an optimized intensity-modulated pelvic radiotherapy technique in patients with prostate cancer. Int J Radiat Oncol Biol Phys.

[18] Guerrero Urbano T, Khoo V, Staffurth J, Norman A, Buffa F, Jackson A, Adams E (2010). Intensity-modulated radiotherapy allows escalation of the radiation dose to the pelvic lymph nodes in patients with locally advanced prostate cancer: preliminary results of a phase I dose escalation study. Clin Oncol (R Coll Radiol).

[19] Muren LP, Wasbo E, Helle SI, Hysing LB, Karlsdottir A, Odland OH, Valen H (2008). Intensity-modulated radiotherapy of pelvic lymph nodes in locally advanced prostate cancer: planning procedures and early experiences. Int J Radiat Oncol Biol Phys.

[20] Fiorino C, Alongi F, Perna L, Broggi S, Cattaneo GM, Cozzarini C, Di Muzio N (2009). Dose-volume relationships for acute bowel toxicity in patients treated with pelvic nodal irradiation for prostate cancer. Int J Radiat Oncol Biol Phys.

[21] Partin AW, Mangold LA, Lamm DM, Walsh PC, Epstein JI, Pearson JD (2001). Contemporary update of prostate cancer staging nomograms (Partin Tables) for the new millennium. Urology.

[22] Krieger A, Susil RC, Menard C, Coleman JA, Fichtinger G, Atalar E, Whitcomb LL (2005). Design of a novel MRI compatible manipulator for image guided prostate interventions. IEEE Trans Biomed Eng.

[23] Shih HA, Harisinghani M, Zietman AL, Wolfgang JA, Saksena M, Weissleder R (2005). Mapping of nodal disease in locally advanced prostate cancer: rethinking the clinical target volume for pelvic nodal irradiation based on vascular rather than bony anatomy. Int J Radiat Oncol Biol Phys.

[24] Cox JD, Stetz J, Pajak TF (1995). Toxicity criteria of the Radiation Therapy Oncology Group (RTOG) and the European Organization for Research and Treatment of Cancer (EORTC). Int J Radiat Oncol Biol Phys.

[25] Gallagher MJ, Brereton HD, Rostock RA, Zero JM, Zekoski DA, Poyss LF, Richter MP (1986). A prospective study of treatment techniques to minimize the volume of pelvic small bowel with reduction of acute and late effects associated with pelvic irradiation. Int J Radiat Oncol Biol Phys.

[26] Bolla M, Gonzalez D, Warde P, Dubois JB, Mirimanoff RO, Storme G, Bernier J (1997). Improved survival in patients with locally advanced prostate cancer treated with radiotherapy and goserelin. N Engl J Med.

[27] Ataman F, Zurlo A, Artignan X, van Tienhoven G, Blank LE, Warde P, Dubois JB (2004). Late toxicity following conventional radiotherapy for prostate cancer: analysis of the EORTC trial 22863. Eur J Cancer.

[28] Seaward SA, Weinberg V, Lewis P, Leigh B, Phillips TL, Roach M (1998). Improved freedom from PSA failure with whole pelvic irradiation for high-risk prostate cancer. Int J Radiat Oncol Biol Phys.

[29] Vargas CE, Galalae R, Demanes J, Harsolia A, Meldolesi E, Nurnberg N, Schour L (2005). Lack of benefit of pelvic radiation in prostate cancer patients with a high risk of positive pelvic lymph nodes treated with high-dose radiation. Int J Radiat Oncol Biol Phys.

[30] Aizer AA, Yu JB, McKeon AM, Decker RH, Colberg JW, Peschel RE (2009). Whole pelvic radiotherapy versus prostate only radiotherapy in the management of locally advanced or aggressive prostate adenocarcinoma. Int J Radiat Oncol Biol Phys.

[31] Pan CC, Kim KY, Taylor JM, McLaughlin PW, Sandler HM (2002). Influence of 3D-CRT pelvic irradiation on outcome in prostate cancer treated with external beam radiotherapy. Int J Radiat Oncol Biol Phys.

[32] Perez CA, Michalski J, Brown KC, Lockett MA (1996). Nonrandomized evaluation of pelvic lymph node irradiation in localized carcinoma of the prostate. Int J Radiat Oncol Biol Phys.

[33] Lawton CA, DeSilvio M, Roach M, Uhl V, Kirsch R, Seider M, Rotman M (2007). An update of the phase III trial comparing whole pelvic to prostate only radiotherapy and neoadjuvant to adjuvant total androgen suppression: updated analysis of RTOG 94-13, with emphasis on unexpected hormone/radiation interactions. Int J Radiat Oncol Biol Phys.

[34] Pommier P, Chabaud S, Lagrange JL, Richaud P, Lesaunier F, Le Prise E, Wagner JP (2007). Is there a role for pelvic irradiation in localized prostate adenocarcinoma? Preliminary results of GETUG-01. J Clin Oncol.

